# Author's Reply. RE: The effectiveness of telepsychiatry: thematic review

**DOI:** 10.1192/bjb.2023.83

**Published:** 2023-12

**Authors:** Karrish Devan, Gunjan Sharma

**Affiliations:** Dual Medical Psychotherapy & General Adult Psychiatry Trainee, Royal College of Psychiatrists, UK. Email: karrish.devan@nelft.nhs.uk; Dual Medical Psychotherapy & Forensic Psychiatry Trainee, Royal College of Psychiatrists, UK.

We thank the authors of the response to our thematic review exploring the effectiveness of telepsychiatry. Much time has passed since its publication in 2021, and here in England guidance for clinicians using virtual medicine has become more available. There also appears, at least anecdotally, to be increasing professional use of hybrid models for both work and patient care.

It seems that the response to our review touches upon two issues relating to telepsychiatry: inequality in access and virtual clinical rotations. As we recognised in our paper, much of the literature has traditionally focused on English-speaking and/or more developed countries. This does pose a risk of skewing data and not representing the intersecting needs of the communities served by telepsychiatry. We would urge future researchers to continue to consider this specifically when designing studies or reviewing the literature.

In relation to the virtual clinical rotations, the emerging research does pose interesting questions about a possible future format for education. This harkens back to the purported origins of telepsychiatry in the 1950s, when it was used for long-distance medical student teaching. We do wonder what the risks would be of moving electives or clinical rotations from face-to-face to virtual. Would students be able, for example, to develop understandings of team working and institutional dynamics? Would they too miss out on the formative phenomenological experiences of being with patients and their families?

Telepsychiatry is effective and likely to continue to be present in many healthcare systems around the world. As clinicians, though, we must strive to ensure that future iterations of virtual medicine remain safe for professionals and patients alike.

